# Supplementary material for: COVID-19 vaccine for people who live and work in prisons worldwide: A scoping review
Source: PLoS One. 2022 Sep 9;17(9):e0267070. doi: 10.1371/journal.pone.0267070 (PMC9462803; doi:10.1371/journal.pone.0267070)
Supplement: Attachment — Submitted filename: Response to Reviewers.docx [file pone.0267070.s005.docx]

**Reviewer #1**

| **No.** | **Reviewer’s Feedback** | **Authors’ Response** |
| --- | --- | --- |
| 1 | Separate out the review findings from the commentary. Right now, the article mixes up the scoping review results, the findings and the analysis, almost like an opinion column mixed up with a scoping review.  The review findings should be presented objectively. The analysis and discussion should follow the findings and be clearly distinct in a separate section. Here is one example:  "Our review discovered that only a minority of countries have prioritised people who  live and work in prisons for vaccination.^71,75,77,79^ Many countries have not explicitly  prioritised these individuals as part of their vaccine rollout, even though some  unambiguously recognised the vulnerability of the prison population in official  publications.^71,75,77^ Such inconsistencies are irrational, unethical and discriminatory,  and perpetuate the marginalisation and social exclusion that many imprisoned  people experienced prior to and during their incarceration. Furthermore, addressing  the health needs of imprisoned people is likely to have a positive impact on reducing  reoffending, thus benefiting all of society."  Everything that follows "Such inconsistencies..." belongs in a separate discussion section that you put after the review findings. This kind of change needs to be made throughout, but especially from pages 18 on. | Thank you for the suggestion. We have separated the findings from the commentary to ensure that they are presented objectively. This is undertaken throughout the ‘Discussions’ section, as well as in other parts of the manuscript. |
| 2 | Tone down the conclusions to align with your actual findings. Right now, the conclusions begin with a very strong statement: "People who live and work in prisons are not being prioritised for Covid-19 vaccinations"... Actually that is not exactly what the findings state. It sounds more like you found from the scoping review that there's a lack of data (especially from LMIC), that a small number of countries are prioritising people in prisons, a larger number of countries are not, and that there is a lack of data from many others. Your conclusions will be more compelling if they are clearly and specifically aligned to the findings. | Thank you. We have now revised our conclusion on page 22. |
| 3 | How many countries are we talking about? It's really unclear from the text when you say "a small number of countries" have done something or "a minority of countries" or "some countries" how many we mean. As this is an important point of the article, could you create a table showing which countries have done what and where we have no data? That would make it easier to see where things stand. | The list of countries, as well as the regional coverage of each paper, has already been provided in Appendix 2. However, this was not made clear in the initial manuscript. We have therefore directed readers to consult Appendix 2 to determine the geographical and regional coverage for each paper in the ‘Methodology’ section.  Additionally, we have attempted to be specific regarding the countries which the analysis relates to. These amendments were made in our ‘Discussions’ section in the manuscript. |
| 4 | Overall this is important work, and especially important that you link the rights of prisoners, rights of people working in prisons, and rights of marginalized communities. I hope you will be able to turn this around quickly for publication. It might even be worthwhile writing a separate opinion column or editorial where you can really cut loose, freely denounce the human rights violations revealed by the study and not be constrained by the strict format of a scoping review. | Thank you, we appreciate your interest in our work. We will consider writing an opinion or an editorial piece as a way forward. |

**Reviewer #2**

| **No.** | **Reviewer’s Feedback** | **Author’s Response** |
| --- | --- | --- |
| 1 | The conclusion is in accordance with the data presented. But not yet connected with research questions (page 5) and research objectives (page 4). | Thank you for the suggestion. We have ensured that our research aims are clearer (page 4, see our response to Question 2 below too), and that they are aligned with our research question (page 5) and conclusion (page 22). |
| 2 | The objectives of the research are not clear enough or there is not enough explanation. In my view, the purpose of this study is to look at the quality of the literature, not to convey information about COVID-19 vaccination in prisons from the literature reviewed. | We agree that the research aims could have been made clearer. As such, we have revised the aims of our research to capture this (p. 4). |
| 3 | Data analysis is appropriate. The manuscript already has data that provides the required information. The manuscripts are presented in an intelligible fashion. | Thank you for this positive feedback. |
